# 25-Hydroxyvitamin D Concentrations Are Lower in Patients with Positive PCR for SARS-CoV-2

**DOI:** 10.3390/nu12051359

**Published:** 2020-05-09

**Authors:** Antonio D’Avolio, Valeria Avataneo, Alessandra Manca, Jessica Cusato, Amedeo De Nicolò, Renzo Lucchini, Franco Keller, Marco Cantù

**Affiliations:** 1 Laboratory of Clinical Pharmacology and Pharmacogenetics, Amedeo di Savoia Hospital, Department of Medical Sciences, University of Turin, 10126 Turin, Italy; valeria.avataneo@unito.it (V.A.); alessandra.manca@unito.it (A.M.); jessica.cusato@unito.it (J.C.); amedeo.denicolo@unito.it (A.D.N.); 2 Department of Laboratory Medicine EOLAB, Ente Ospedaliero Cantonale, 6500 Bellinzona, Switzerland, Renzo.Lucchini@eoc.ch (R.L.); Franco.Keller@eoc.ch (F.K.); Marco.Cantu@eoc.ch (M.C.)

**Keywords:** vitamin D, SARS-CoV-2, concentrations, COVID-19, coronavirus, deficiency

## Abstract

Severe acute respiratory syndrome coronavirus 2 (SARS-CoV-2) causes coronavirus disease 2019 (COVID-19), with a clinical outcome ranging from mild to severe, including death. To date, it is unclear why some patients develop severe symptoms. Many authors have suggested the involvement of vitamin D in reducing the risk of infections; thus, we retrospectively investigated the 25-hydroxyvitamin D (25(OH)D) concentrations in plasma obtained from a cohort of patients from Switzerland. In this cohort, significantly lower 25(OH)D levels (*p* = 0.004) were found in PCR-positive for SARS-CoV-2 (median value 11.1 ng/mL) patients compared with negative patients (24.6 ng/mL); this was also confirmed by stratifying patients according to age >70 years. On the basis of this preliminary observation, vitamin D supplementation might be a useful measure to reduce the risk of infection. Randomized controlled trials and large population studies should be conducted to evaluate these recommendations and to confirm our preliminary observation.

## 1. Introduction

Severe acute respiratory syndrome coronavirus 2 (SARS-CoV-2) causes coronavirus disease 2019 (COVID-19), with clinical outcomes ranging from mild to severe, including death. To date, there is no specific recommended treatment, with COVID-19- and SARS-COV-2-affected patients targeted to receive supportive care to help relieve symptoms.

However, only a fraction of infected people show clinical symptoms, and an even lower percentage require medical attention [1,2]. To date, it is not yet known why some patients develop more severe symptoms.

Recently, some articles have suggested the possible involvement of vitamin D in reducing the risk of respiratory tract infections, especially in the influenza and COVID-19 context. Furthermore, the role of vitamin D supplementation in reducing the risk of infection [3,4,5,6] is still under investigation, however, no clinical evidence has been reported yet.

For these reasons, we retrospectively described the 25-hydroxyvitamin D (25(OH)D) plasma concentrations in a cohort of patients from Switzerland.

## 2. Methods

### 2.1. Data Collection

We retrospectively evaluated the repository data for patients who underwent a nasopharyngeal swab PCR analysis for SARS-CoV-2 and a 25(OH)D measurement at “Ente Ospedaliero Cantonale” (Canton of Tessin, Switzerland), during the period from 1 March to 14 April 2020.

According to the Swiss federal office for public health’s (Bundesamt für Gesundheit (BAG)) rules, patients selected for the SARS-CoV-2 PCR analysis had to have symptoms of an acute airway disease (e.g., cough, sore throat, breathing difficulties), with or without fever, feeling of fever, muscle pain, or sudden anosmia or ageusia.

The vitamin D analysis was required to be conducted within seven weeks of the SARS-CoV-2 PCR result. As an additional control cohort, all patients with a 25(OH)D measurement during the same period (1 March to 14 April) of 2019 were evaluated.

The duration of sunshine, expressed as total sun hours, into the analyzed period (1 March to 14 April) were compared between 2019 and 2020. Data were provided by the federal office of meteorology and climatology MeteoSwiss. Reported data refer to the Locarno-Monti measuring station.

### 2.2. Sample Processing for Vitamin D Quantitation

Samples were processed with MassChrom^®^ 25-OH-Vitamin D3/D2 in serum/plasma (Chromsystems, Germany) on a MassStar liquid handler (Hamilton, Switzerland) according to the manual’s procedures. The extracted samples analysis was carried out with a liquid chromatography coupled with a tandem mass spectrometry (LC-MS/MS) instrument G6490A (Agilent Technologies, Santa Clara, CA, USA) equipped with a 1290 Infinity LC Systems UHPLC.

### 2.3. PCR Analysis for COVID-19 Identification

Samples were collected using the Copan^®^ FLOQSwabs^®^ UTM^®^ Nasopharyngeal Sample Collection Kit composed of the Flexible Minitip Flock Swab + 3 mL UTM^®^ Viral Transport Medium (COPAN, Italy). An amount of 200 µL of media was extracted with the MagPurix^®^ Viral/Pathogen Nucleic Acid Extraction Kit B using an Automated Nucleic Acid Purification System MagPurix 12s (Zinexts, Taiwan) and amplified with a Sars-COV (COVID19) E-gene and RdRp Gene kit (TIB MOLBIOL, Germany). Data acquisition was performed with an ABI 7500 Fast Real-Time PCR (Applied Biosystems, Foster City, CA, USA).

### 2.4. Statistical Analysis

For the descriptive statistics, the continuous variables are summarized as the median (25th–75th percentile, interquartile range (IQR)). The categorical variables are described as frequencies and percentages. All data were assessed for normality using a Shapiro–Wilk test and the categorical data were compared using Mann–Whitney or Kruskal–Wallis statistical tests.

Spearman’s rank correlation was utilized to determine the continuous data.

Statistical analyses were carried out using the SPSS software package, version 26.0 (IBM, Armonk, NY, USA).

## 3. Results

The 2020 cohort of 107 total patients (male = 54.2%; median age = 73 years (IQR 63–81); median 25(OH)D = 22.0 ng/mL (IQR 8.9–30.5)) included 27 SARS-CoV-2 PCR-positive (male = 70.4%; median age = 74 years (IQR 65–81) with median 25(OH)D = 11.1 ng/mL (IQR 8.2–21.0)), and 80 SARS-CoV-2 PCR-negative (male = 48.8%; median age = 73 years (IQR 61–82) with median 25(OH)D = 24.6 ng/mL (IQR 8.9–30.5)) patients. The measurement of 25(OH)D was generally performed three days after the molecular PCR test (overall median days away = −3.0 (IQR −7.0–0.0)), and a not statistically significant difference in days away was found: −2.0 days (IQR −7.0–1.75) vs. −3.0 (IQR −6.0 to −1.0) (*p* = 0.119) within the PCR-negative and PCR-positive patients, respectively.

As an additional control cohort, without a SARS-CoV-2 PCR test, all patients with at least one measurement for 25(OH)D in the corresponding period (1 March to 14 April) of 2019 (before Covid-19 pandemic) were evaluated, with a total of 1377 patients (male = 45.3%; median age = 63 years (IQR 46–76); median 25(OH)D = 24.6 ng/mL (IQR 16.2–33.0)). The total sun hours in the analyzed period (1 March to 14 April) are 333.4 h and 349.4 h in 2019 and 2020, respectively, with a total increment of 16 h in 2020 (+4.8%).

**Figure 1:**
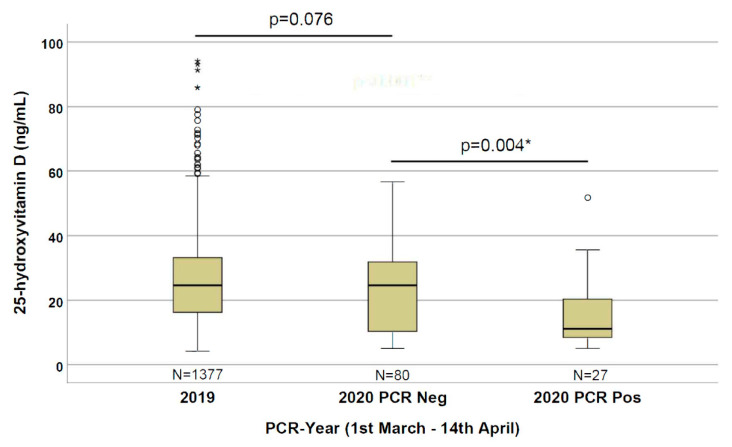
25-hydroxyvitamin D concentrations in the three evaluated groups (patients from 1 March to 14 April of 2019 and 2020 with a negative PCR, and of 2020 with a positive PCR for SARS-CoV-2. *: significant results.

As depicted in Figure 1, we observed statistically significant (*p* = 0.004) lower 25(OH)D levels (11.1 ng/mL) in patients positive for the SARS-CoV-2 PCR compared with the negative patients (24.6 ng/mL). By comparing the 2020 and 2019 cohorts, we observed an even stronger statistically significant difference (*p* < 0.001) in 25(OH)D levels in patients with a positive PCR for SARS-CoV-2 compared with the 2019 patients (24.6 ng/mL); however, no significant difference (*p* = 0.076) between the 2019 and 2020 negative PCR cohorts was observed.

**Figure 2:**
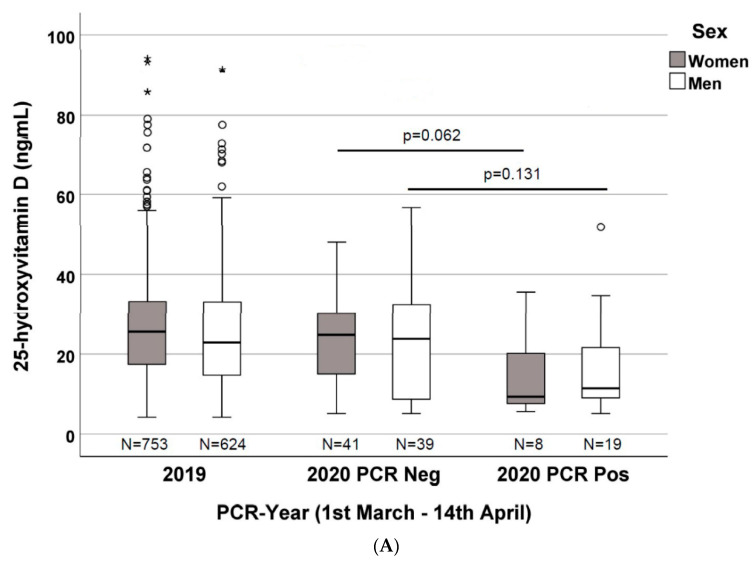

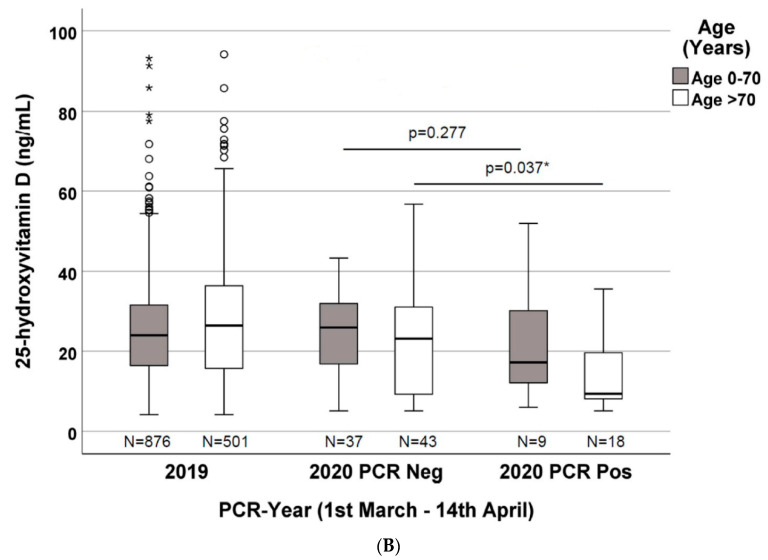
(**A**) 25-hydroxyvitamin D concentrations in the three evaluated groups divided by gender (patients from 1 March to 14 April of 2019 and 2020 with a negative PCR, and of 2020 with a positive PCR to SARS-CoV-2; (**B**) 25-hydroxyvitamin D concentrations in the three evaluated groups divided by age (0–70 years vs. >70 years) (patients from 1 March to 14 April of 2019 and 2020 with a negative PCR, and of 2020 with a positive PCR for SARS-CoV-2. *: significant results.

As depicted in Figure 2A, when dividing the 2020 cohort according to gender and PCR result, a non-statistically significant difference in vitamin D concentrations was found: 24.8 ng/mL (IQR 14.5–30.9) vs. 9.3 ng/mL (IQR 7.3–20.5) (*p* = 0.062) and 23.8 ng/mL (IQR 7.13–32.7) vs. 11.4 ng/mL (IQR 8.9–23.6) (*p* = 0.131) within women (41 vs. 8) and men (39 vs. 19), respectively. Nevertheless, vitamin D concentrations were significantly different when comparing patients from the 2019 and 2020 PCR-positive cohorts, stratified by gender: 25.6 ng/mL (IQR 17.3–33.3) vs. 9.3 ng/mL (IQR 7.3–20.5) (*p* = 0.019) in women (*n* = 753 vs. *n* = 8) and 22.9 ng/mL (IQR 14.7–33.1) vs. 11.4 ng/mL (IQR 8.9–23.6) (*p* = 0.005) in men (*n* = 624 vs. *n* = 19).

As depicted in Figure 2B, when stratifying the 2020 patients by age (0–70 years and >70 years) and PCR positivity, the vitamin D concentrations are not significantly different (*p* = 0.277) among the two groups (*n* = 37 vs. *n* = 9), with median values of 25.9 ng/mL (IQR 15.9–32.1) vs. 17.2 ng/mL (IQR 11.7–31.6), respectively. Nevertheless, when considering only patients with age >70 years (*n* = 43 vs. *n* = 18), the vitamin D concentrations are significantly different (*p* = 0.037), with median values of 23.1 ng/mL (IQR 8.5–31.7) in PCR-negative patients vs. 9.3 ng/mL (IQR 8.1–19.9) in PCR-positive patients. Moreover, when comparing patients enrolled in 2019 with the 2020 PCR-positive patients, the vitamin D concentrations are even more significantly different (*p* < 0.001): in patients with age >70 years (*n* = 501 vs. *n* = 18), the median was 26.4 ng/mL (IQR 15.7–36.4) in the 2019 cohort vs. 9.3 ng/mL (IQR 8.1–19.9) in the 2020 cohort. The same difference was not observed when comparing patients with an age lower than 70 years (*n* = 876 vs. *n* = 9), with median values of 23.9 ng/mL (IQR 16.4–31.6) vs. 17.2 ng/mL (IQR 11.7–31.6) (*p* = 0.287), respectively.

## 4. Discussion

The world is in the grip of the COVID-19 pandemic. Public health measures to reduce the risk of infection and death, in addition to quarantines, are desperately needed. In this paper, we describe for the first time that the 25(OH)D level is significantly lower in SARS-CoV-2 PCR-positive patients than in PCR-negative patients (Figure 1).

Despite the relatively low numbers, this evidence is particularly strong, since the group of PCR-negative patients had a risk of infection and symptoms of respiratory tract infections as indications for the PCR testing. Since the risk of symptomatic upper respiratory tract infection is suggested to be associated with low 25(OH)D levels [7], its concentration is expected to be quite low in PCR-negative patients (partially confirmed by the trend of a difference with the 2019 cohort, *p* = 0.076), making this control group even more stringent. Therefore, the significantly lower 25(OH)D concentrations in the PCR-positive group could indicate that the risk of SARS-CoV-2 infection has a stronger relationship with the 25(OH)D concentration, rather than other respiratory tract infections.

Nevertheless, no precise information regarding the clinical conditions of the PCR-negative patients was available in this study, making further stratification impossible.

On the other hand, when stratifying the 2020 patients by gender and PCR positivity, the vitamin D concentrations were different, with a clear trend, but were not statistically significant, probably due to the low number in each cluster. In fact, when we consider the 2019 patients, the vitamin D concentrations were significantly different for both the 2020 PCR-positive women (753 vs. 8) and men (624 vs. 19), respectively (Figure 2A).

In our cohort, the 25(OH)D concentrations were significantly lower in patients with a positive SARS-CoV-2 PCR assay when compared with the PCR-negative patients and patients from the 2019 cohort, when the age was >70 years (Figure 2B). There were no differences when we compared patients with an age <70 years. These data may be important if we consider that age is a well-known predictor of disease severity in COVID-19. Based on this evidence, we could hypothesize that higher 25(OH)D levels (perhaps around 30 ng/mL as a possible target) could reduce the risk if severe disease in the elderly (>70 years).

Our data have several possible biases: the data represented a relatively low number of patients from a single hospital center, no clinical information was available about the severity of COVID-19 symptoms for PCR-positive patients, no clinical information was available about symptoms in PCR-negative patients (without a possible clinical stratification), etc. Moreover, other potential confounding variables linking SARS-CoV-2 PCR positivity and lower vitamin D levels could be diet or possible vitamin D supplementation (data not available). Finally, the PCR for SARS-CoV-2 and the 25(OH)D quantification could be performed on different days, but the median days away were close in both the PCR-positive and PCR-negative groups, with no statistical difference between them. Therefore, the half-life of 25(OH)D (approximately 2–3 weeks) should not influence our results.

The exposure to the sun could be important. The total sun hours in the analyzed period (1 March to 14 April) were 333.4 h and 349.4 h in 2019 and 2020, respectively. The difference in sunshine was 4.8% and it seems to be not significant. Eating behavior and the possible vitamin D supplementation are more likely to be important factors to consider [3]. Another critical issue that we could consider is the possible reverse causality, where patients with COVID-19 could have a drop in their 25(OH)D levels due to the SARS-CoV-2 infection. However, with a relatively short disease, this issue can be considered unlikely and no data are available. In conclusion, this study represents a preliminary observation justified by several described mechanisms through which 25(OH)D can reduce the risk of infections [3]. These mechanisms include the induction or transcription of cathelicidins and defensins that can reduce viral replication rates and concentrations of pro-inflammatory cytokines responsible for producing inflammation and injuring the lining of lungs, leading to pneumonia, as well as the capability of vitamin D to increase the concentrations of anti-inflammatory cytokines. Several pieces of evidence support the role of vitamin D in reducing the risk of COVID-19, including, as indicated by other authors, that the outbreak occurred in winter, a time when 25(OH)D concentrations are low; that the number of cases in the Southern Hemisphere near the end of summer is low; that vitamin D deficiency has been found to contribute to acute respiratory distress syndrome [3]; and that case fatality rates increase with age (>70 years) and with chronic disease comorbidity, both of which are associated with a lower 25(OH)D concentration [3].

As suggested by Grant et al., it is recommended that people at risk of COVID-19 consider taking 10,000 IU/day of vitamin D3 for a few weeks to rapidly increase their 25(OH)D concentrations, followed by 5000 IU/day to reduce the risk of infection. The goal should be to raise 25(OH)D concentrations above 40–60 ng/mL (100–150 nmol/L) [3], or at least 30 ng/mL, considering our preliminary data.

It is probable that vitamin D3 supplementation would be useful in the treatment of COVID-19 infection, in preventing a more severe symptomatology and/or in reducing the presence of the virus in the upper respiratory tract and making the patients less infectious (justifying negative PCR in people with higher 25(OH)D). Randomized controlled trials and large population studies should be conducted to evaluate these recommendations and to confirm our preliminary observations and hypothesis.
